# Welfare states, the Great Recession and health: Trends in educational inequalities in self-reported health in 26 European countries

**DOI:** 10.1371/journal.pone.0193165

**Published:** 2018-02-23

**Authors:** Teresa Leão, Inês Campos-Matos, Clare Bambra, Giuliano Russo, Julian Perelman

**Affiliations:** 1 Escola Nacional de Saúde Pública, Universidade NOVA de Lisboa, Lisboa, Portugal; 2 Instituto de Higiene e Medicina Tropical, Universidade NOVA de Lisboa, Lisboa, Portugal; 3 Centro de Investigação em Saúde Pública, Universidade NOVA de Lisboa, Lisboa, Portugal; 4 Travel and Migrant Health section, National Infection Service, Public Health England, London, United Kingdom; 5 Institute for Health and Society, Newcastle University, Newcastle, United Kingdom; 6 Centre for Primary Care and Public Health, Queen Mary University of London, London, United Kingdom; Universidad de Castilla-La Mancha, SPAIN

## Abstract

**Background:**

Although socioeconomic inequalities in health have long been observed in Europe, few studies have analysed their recent patterning. In this paper, we examined how educational inequalities in self-reported health have evolved in different European countries and welfare state regimes over the last decade, which was troubled by the Great Recession.

**Methods:**

We used cross-sectional data from the EU-SILC survey for adults from 26 European countries, from 2005 to 2014 (n = 3,030,595). We first calculated education-related absolute (SII) and relative (RII) inequalities in poor self-reported health by country-year, adjusting for age, sex, and EU-SILC survey weights. We then regressed the year- and country-specific RII and SII on a yearly time trend, globally and by welfare regime, adjusting for country fixed effects. We further adjusted the analysis for the economic cycle using GDP growth, unemployment, and income inequality.

**Results:**

Overall, absolute inequalities persisted and relative inequalities slightly widened (beta_RII_ = 0.0313, p<0.05). There were substantial differences by welfare regime: Anglo-Saxon countries experienced the largest increase in absolute inequalities (beta_SII_ = 0.0032, p<0.05), followed by Bismarkian countries (beta_SII_ = 0.0024, p<0.001), while they reduced in Post-Communist countries (beta_SII_ = -0.0022, p<0.001). Post-Communist countries also experienced a widening in relative inequalities (beta_RII_ = 0.1112, p<0.001), which were found to be stable elsewhere. Adjustment for income inequality only explained such trend in Anglo-Saxon countries.

**Conclusions:**

Educational inequalities in health have overall persisted across European countries over the last decade. However, there is considerable variation across welfare regimes, possibly related to underpinning social safety nets and to austerity measures implemented during this 10-year period.

## Introduction

There is substantial evidence in Europe that individuals from lower educated groups experience a higher risk of mortality,[1] a higher prevalence of chronic diseases,[1,2] and poorer self-reported health (SHR).[1,3,4] Lower educational status is usually associated with lower health literacy[5,6], worse working conditions, lower income and precarious living conditions[6] that affect physical and mental health.[6,7]

Though such health inequalities (HI) are observed across all European countries,[1–4] societies with strong social protection and high social cohesion are expected to mitigate the impact of unfavourable socioeconomic circumstances.[6–9] Countries with more redistributive social security systems, universal education and health coverage tend to experience better health amongst all socio-economic groups, although there is less clear evidence about the magnitude of absolute and relative HI.[10] However, it is much more firmly established that countries with lower social benefits, fragmented welfare provision and partial health and educational coverage, are characterized by the highest HI in Europe.[1,8,9,11,12] Furthermore, the ‘cushion effect’ of the universalist and encompassing welfare state is believed to be triggered especially during times of economic crisis, by protecting the most vulnerable from the effects of unemployment and income reduction.[6,12,13] Therefore, societies with weaker social safety nets are not only expected to have higher HI, but that these would also widen during crisis periods.

Data from the 1980s and 1990s showed that HI have persisted in Europe for the last 40 years,[3,4,14,15] even in countries with strong social protection regimes. However, few studies have analysed the evolution of HI during the last decade,[3,14,15] which was marked by the ‘Great Recession’. Recessions are defined as two successive quarters of negative growth in GDP. They are characterised by instability and sudden reductions in production and consumption, with corresponding increases in unemployment. The post-2008 economic downturn is popularly referred to as the ‘Great Recession’ as it has been longer, wider and deeper than any previous economic downturns, including the ‘Great Depression’ of the 1930s.[16] It affected all European countries to varying degrees and with different policy responses. Some countries, particularly those in southern Europe and the UK, pursued policies of austerity, whilst others maintained their social protection regimes such as Norway and Sweden. ‘Austerity’ refers to reducing budget deficits in economic downturns by decreasing public expenditure, particularly on welfare, and/or increasing taxes.[16] It is expected therefore that the evolution of HI in these divergent policy regimes would differ, with those pursuing austerity potentially putting the health of their most vulnerable groups at risk,[16–18] leading to widening HI.[6]

In order to better understand how HI evolved across Europe during this period and which welfare regimes were better at preventing increases in the health gap, we: (i) examined the evolution of HI across 26 European countries from 2005 to 2014; and (ii) compared this evolution by welfare state regime type taking into account these countries’ economic cycles.

## Methods

### Data

We used repeated cross-sectional individual data from the European Union Statistics on Income and Living Conditions (EU-SILC) database.[19] We included all countries with yearly data available from 2005 to 2014 at the moment we started the study (Austria, Belgium, Cyprus, Czech Republic, Denmark, Germany, Greece, Estonia, Spain, Finland, France, Hungary, Ireland, Iceland, Italy, Latvia, Lithuania, Luxembourg, Netherlands, Norway, Poland, Portugal, Sweden, Slovenia, Slovak Republic, and United Kingdom).

We only considered adults aged 25 to 79 years old; we excluded people younger than 25 as most of them might not have concluded their education (our measure of socioeconomic status) (556,938 observations excluded, 12.79% of the sample), and people over 79 because the EU-SILC sample does not include people living in collective households and institutions[19] (215,670 observations excluded, 4.95% of the sample). The inclusion of this latest group could introduce a bias[3] since institutionalized groups tend to have a worse health status than not institutionalized groups[20], and the proportion of institutionalized people varies from country to country[21]. Moreover, EU-SILC codes all respondents over eighty years-old as having 80 or 81 years-old, which would hinder age standardization.[3] Observations with missing data on SRH were also excluded (550,165 observations, i.e., 15.4% of cases). The final sample included 3,030,595 observations. The number of observations per country-year ranged from 2,316 (Iceland, 2006) to 39,843 (Italy, 2005).

‘Poor SRH’ was selected as dependent variable. This variable has been considered as a reliable proxy of health status and mortality[22,23] and has been widely used for the measurement of HI.[3,4,22,24] Five items (‘very good’, ‘good, ‘fair’, ‘bad’ and ‘very bad’) were available to answer the question ‘How is your health in general?’. We grouped the ‘bad’ and ‘very bad’ answers into the ‘poor SRH’ category.

We stratified the population by education level. This is a stable measure of socioeconomic status across life, less subject to reverse causation than other measures (such as income), easily comparable across countries, and, thus, widely used to measure HI.[2–4,8,14,15,25] We classified the seven International Standard Classification of Education categories of the highest level attained into three groups: ‘1’–no education, pre-primary, primary and lower secondary education; ‘2’–upper secondary and post-secondary education; and ‘3’–tertiary education.[3] This simplification allowed the comparison of HI across countries, while ensuring enough observations per education category in each country/year sub-group.

We included sex and age as covariates. Age was included as a continuous variable.

In order to understand how the social welfare context may influence HI, we followed the welfare state typology most frequently used in public health studies,[9–11,26–30] and grouped the 26 countries into six welfare regimes: Scandinavian, Southern, Bismarckian, Anglo-Saxon, Former USSR, and Post-communist. These are described in Table 1.

**Table 1:** Organization and description of European welfare state regimes [9–11,26–31].

**Scandinavian:** Denmark, Finland, Iceland, Norway, and Sweden.
Characterised by universalism, comparatively generous social transfers, a commitment to full employment and income protection; and a strongly interventionist state. The state is used to promote social equality through a redistributive social security system. Unlike the other welfare state regimes, the Scandinavian regime type promotes an equality of the highest standards, not an equality of minimal needs and it provides highly decommodifying programs.
**Bismarckian:** Austria, Belgium, Germany, France, Luxembourg, and the Netherlands.
Distinguished by its ‘status differentiating’ welfare programs in which benefits are often earnings related, administered through the employer; and geared towards maintaining existing social patterns. The role of the family is also emphasised and the redistributive impact is minimal. However, the role of the market is marginalised.
**Anglo-Saxon:** UK and Ireland.
State provision of welfare is minimal, social protection levels are modest and often attract strict entitlement criteria; and recipients are usually means-tested and stigmatised. In this model, the dominance of the market is encouraged both passively, by guaranteeing only a minimum, and actively, by subsidising private welfare schemes. The Anglo-Saxon welfare state regime thereby minimises the decommodification effects of the welfare state and a stark division exists between those, largely the poor, who rely on state aid and those who are able to afford private provision.
**Southern:** Cyprus, Spain, Greece, Italy, and Portugal.
The southern welfare states have been described as ‘rudimentary’ because they are characterised by the smallest public expenditure per capita in social protection in western Europe, and the highest per capita out-of-pocket expenditures on health. Their welfare provision consists of diverse income maintenance schemes that range from the meagre to the generous and welfare services, particularly the health care system. Reliance on the family is a prominent feature.
**Post-communist:** Czech Republic, Hungary, Poland, Slovenia, and Slovakia.
The formerly Communist countries of Eastern Europe have experienced the demise of the universalism of the Communist welfare state and a shift towards policies associated more with the Anglo-Saxon (marketization and decentralisation) regime—including financing via taxation rather than insurance contributions. They are characterized by lower than average EU social expenditure but with lower income inequalities and higher social well-being than the Former USSR countries.
**Former-USSR**: Estonia, Lithuania, and Latvia.
These countries are characterized by low public spending on social programs, which are mainly financed through social insurance contributions echoing the Bismarckian (social insurance) regime.

We further considered unemployment rate, the annual percentage growth of the Gross Domestic Product (GDP), and the Gini index (of income inequalities) as indicators of economic cycles. GDP growth measures the national productivity, which may not be sufficient to capture the unequal effects of the Great Recession and the policy responses to it. This is why we completed this indicator by unemployment and income inequality measures, used as complementary means to address the possible effects of the Great Recession. Yearly and per country GDP growth, unemployment rates and Gini index were obtained from the World Bank[32] and Eurostat[33] databases, respectively.

### Statistical methods

We first calculated HI among educational groups, stratifying the observations per year (globally), welfare-year, and country-year units (S1 Table). We used absolute (slope index of inequality, SII) and relative measures (relative index of inequality, RII), which allow to account for the distribution of health status across all socioeconomic groups, while taking into account the distribution of the population across socioeconomic status.[24,34,35] SII may be interpreted as the difference in rates between the two extremes of the education hierarchy, and allows observe the impact of a policy in absolute terms, which is more relevant for public health (e.g., number of cases prevented). As SII is sensitive to changes of the prevalence of a certain condition on a population, [34,35] it must be complemented by the RII. That is, when the prevalence lowers, absolute variations become smaller but still relevant, calling for using variation measures in relative terms. The RII can be interpreted as the ratio of the morbidity between the least and most educated groups, and it is relevant to assess the impact of a reform or intervention in low-prevalence contexts. As both measures take into account the distribution of health among intermediate groups and their dimension, their rise can be attributed to a widening of health inequalities across education groups, and/or to a widening of the distribution of the population across education groups[34,35]. Because these two measures behave differently in high and low prevalent contexts, they must be complemented to better express the impact of the macroeconomic and policy changes on HI.[34,35]

**Fig 1:**
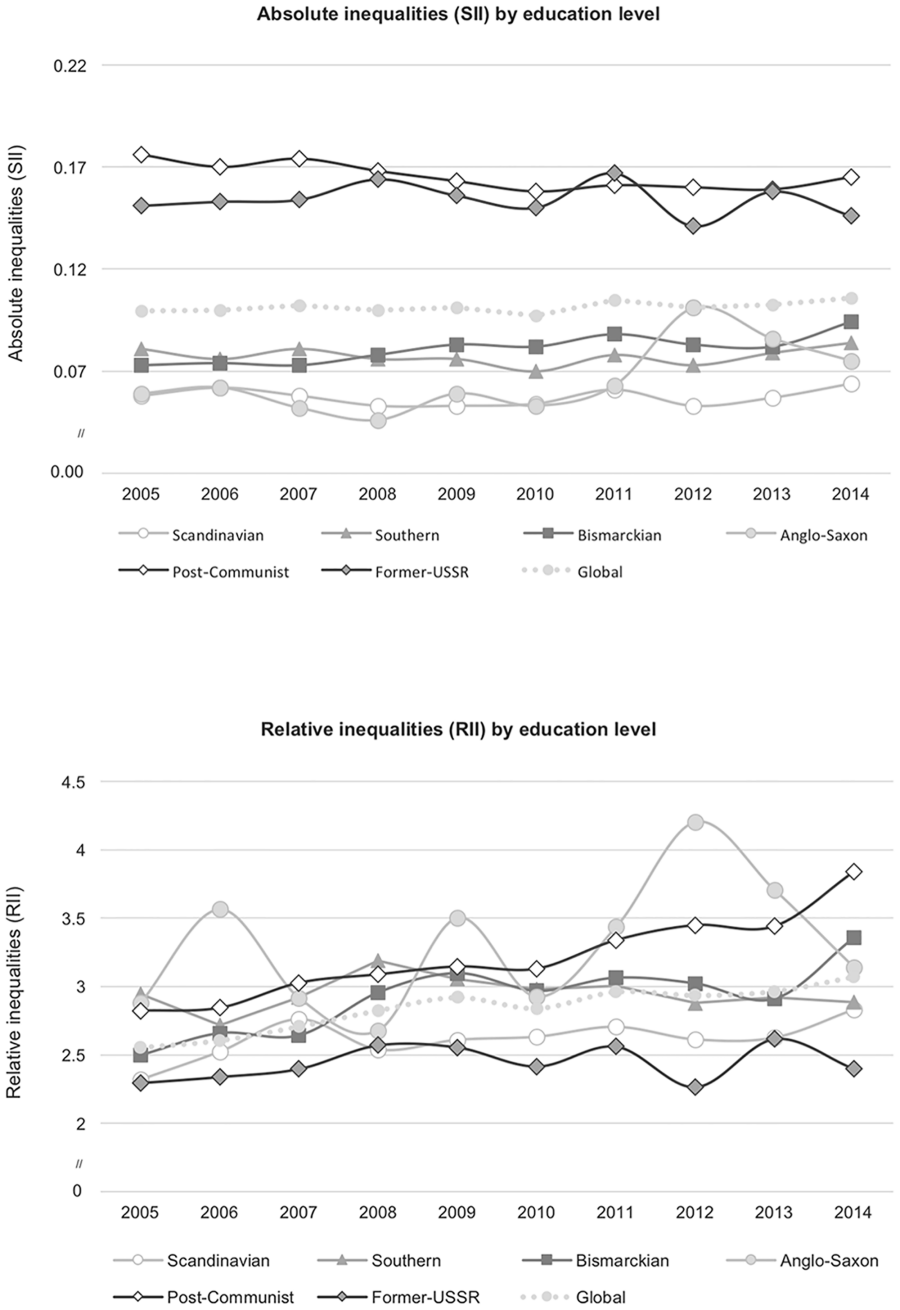
Evolution of health inequalities between 2005 and 2014. Absolute (SII) and relative (RII) inequalities on poor SRH plotted by year, globally and by social welfare regime (adjusted for sex, age and country fixed effects).

Country-year SII and RII were calculated controlling for age, and sex. Personal cross sectional weights, provided by the EU-SILC database,[19] were used to adjust for non-response. Global- and welfare-year SII and RII were further adjusted for country fixed effects, and were plotted in Fig 1 and in S1 Fig.

Secondly, we created a database compiling the 260 country-year calculated RII and SII. In order to estimate HI time trends, we stratified the countries per welfare, and regressed these country-year RII and SII on a yearly time trend, adjusting for country fixed effects (Model 1, below). Finally, to test whether variations in HI were explained by economic cycles, we additionally adjusted for GDP growth, unemployment, Gini index, and all three proxies together (Models 2, 3, 4, and 5, respectively).

Model 1: HI*ct* = *α* + *Trendt* + *Countryc* + *ε*
Model 2: HI*ct* = *α* + *Trendt* + *Countryc* + *GDPct* + *ε*
Model 3: HI*ct* = *α* + *Trendt* + *Countryc* + *UNEMPct* + *ε*
Model 4: HI*ct* = *α* + *Trendt* + *Countryc* + *GINIct* + *ε*
Model 5: HI*ct* = *α* + *Trendt* + *Countryc* + *GDPct* + *UNEMPct* + *GINIct* + *ε*

where ‘HI’ is either RII or SII, that is, these five equations were estimated for both SII and RII indicators. The indices ‘t’ and ‘c’ refer to the year and country, respectively. The ‘Country’ variable stands for country fixed effects, and the ‘Trend’ for the yearly linear trend. These models were estimated globally and then by the six welfare regimes, so we finally ran 5x2x(1+6) = 70 regressions.

## Results

The descriptive analysis showed population aging in all welfare regimes and increasing proportion of individuals with tertiary education over the decade, except in Bismarckian countries (Table 2). The prevalence of poor SRH globally decreased over this 10-year period except in Anglo-Saxon countries, where it increased from 2008 on, exceeding the 2005 value in 2014. Southern and Post-Communist countries experienced the lowest tertiary education rates, with a disadvantage of 15 percentage points (pp) compared to Anglo-Saxon and Scandinavian countries. Former-USSR and Post-Communist countries displayed the highest prevalence of poor SRH.

**Table 2:** Characteristics of the sample, per welfare regime and year (weighted). All values represent percentages.

	2005	2006	2007	2008	2009	2010	2011	2012	2013	2014
**All countries**										
Female	51.87	51.90	51.83	51.89	51.94	51.86	51.71	51.82	52.13	52.05
Older than 55	37.59	37.34	37.79	37.90	38.42	38.66	39.01	39.20	40.00	40.36
Tertiary education	24.34	21.68	21.68	23.51	23.92	24.81	25.71	26.66	27.07	27.62
Poor SRH	10.93	10.68	10.38	9.26	9.23	8.90	9.19	9.36	9.20	9.09
Sample size	297,176	304,230	308,572	306,968	304,091	303,543	302,647	308,270	295,786	299,312
**Scandinavian**										
Female	50.76	51.25	50.85	50.69	50.67	50.55	50.06	50.29	49.43	49.92
Older than 55	39.07	39.97	40.64	41.19	41.00	42.29	42.58	42.84	42.97	43.02
Tertiary education	28.43	29.48	29.68	30.69	31.65	32.48	33.32	36.66	36.26	39.01
Poor SRH	7.93	8.33	7.55	7.36	7.06	6.88	7.14	6.69	6.51	6.35
Sample size	26,284	25,376	25,875	26,570	26,302	26,613	23,920	25,484	26,171	26,930
**Southern**										
Female	51.12	51.07	50.97	50.89	50.82	50.76	50.81	51.06	51.12	51.16
Older than 55	36.73	36.36	36.42	36.25	36.39	36.45	36.77	37.50	38.01	38.39
Tertiary education	17.16	17.52	18.25	19.20	19.64	19.98	20.35	21.06	22.01	23.20
Poor SRH	10.91	10.89	10.74	9.37	9.24	8.49	9.70	9.47	9.61	9.26
Sample size	91,116	87,719	85,693	87,721	88,322	87,053	84,735	85,025	85,056	89,003
**Bismarckian**										
Female	51.71	51.84	51.80	51.83	51.68	51.59	51.60	51.67	51.63	51.68
Older than 55	39.31	38.91	39.66	39.86	40.40	40.49	41.11	41.01	41.42	41.73
Tertiary education	30.65	23.13	24.29	24.66	25.29	25.86	26.73	27.67	28.29	28.38
Poor SRH	8.96	8.99	8.88	7.81	8.05	8.13	8.17	8.17	7.88	7.84
Sample size	67,325	71,690	75,934	72,264	72,237	74,490	76,460	77,409	70,997	71,009
**Anglo-Saxon**										
Female	52.98	52.79	52.63	53.06	53.15	52.84	51.66	51.84	54.51	53.89
Older than 55	37.37	37.00	37.25	36.82	37.03	37.59	36.96	36.40	39.10	39.89
Tertiary education	30.97	30.99	25.04	34.48	33.79	36.57	37.86	39.20	39.55	38.78
Poor SRH	7.03	6.30	6.04	4.90	5.47	5.78	5.74	7.89	7.76	8.70
Sample size	24,910	23,210	21,995	20,689	19,080	18,079	18,344	21,316	19,210	20,372
**Post-Communist**										
Female	52.73	52.80	52.79	52.81	53.83	53.90	53.95	53.68	53.96	53.54
Older than 55	34.62	34.90	35.57	36.14	37.73	38.27	38.99	39.42	40.19	40.34
Tertiary education	14.85	15.75	16.47	17.29	17.92	19.01	19.84	20.88	21.66	22.25
Poor SRH	18.02	17.55	16.56	15.86	14.64	14.13	13.89	13.32	12.75	15.64
Sample size	65,967	70,975	73,971	76,455	73,662	72,110	73,409	73,407	69,131	67,688
**Former-USSR**										
Female	55.07	55.04	55.09	57.44	57.25	57.48	56.88	57.56	57.77	57.21
Older than 55	36.92	36.67	36.64	38.07	39.15	39.49	39.36	40.27	40.73	41.35
Tertiary education	22.70	23.98	24.84	26.66	27.15	28.58	29.29	30.28	31.62	31.60
Poor SRH	20.19	18.53	16.71	17.03	16.64	16.78	17.37	16.77	16.10	15.57
Sample size	21,574	25,260	25,104	23,269	24,488	25,198	25,779	25,629	25,221	24,310

In 2005, the least educated groups had a 9.96 pp higher prevalence (SII) and 2.56 higher odds (RII) of having poor SRH (Fig 1 and S1 Fig). Across this decade, Former-USSR countries, together with Post-Communist, presented the highest absolute inequalities: least educated groups experienced between 14–18 pp higher prevalence of poor SRH than the highest-educated groups, while in all other welfare regimes this difference was smaller than 10 pp. By contrast, RII was higher among Post-Communist and Southern countries, except in 2006, 2009, 2012, when the Anglo-Saxon sample displayed substantial peaks.

Globally, absolute inequalities persisted over this 10-year period (Tables 3 and 4). When stratified by welfare regime, an increase in SII was observed in Anglo-Saxon (beta = 0.0032, p<0.05) and Bismarckian (beta = 0.0024, p<0.001) countries, and a decrease in Post-Communist countries (beta = -0.0022, p<0.001). No significant trend was observed for the other welfare regimes. The slight widening of global RII (beta = 0.0313, p<0.05) was preponderantly driven by the positive and statistically significant yearly trend in Post-communist countries (beta = 0.1112, p<0.001). The other welfare regimes did not experience major changes in RII.

**Table 3:** Evolution of absolute (SII) inequalities from 2005 to 2014, adjusting for GDP growth, unemployment, and Gini index.

	Scandinavian	Southern	Bismarckian	Anglo-Saxon	Post-communist	Former-USSR	Global
**SII** [95% CI]							
**Model 1**							
Time trend (yearly)	0.0005 [-0.0008;0.00175]	-0.0006 [-0.019;0.0006]	0.0024*** [0.0016;0.0032]	0.0032* [0.0007;0.0057]	-0.0022*** [-0.0032;-0.0011]	-0.0003 [-0.0025;0.0018]	0.0003 [-0.0024;0.0009]
**Model 2**							
Time trend (yearly)	0.0008 [-0.0004;0.0021]	-0.0001 [-0.0015;0.0014]	0.0025*** [0.0016;0.0033]	0.0032* [0.0007;0.0057]	-0.0016** [-0.0028;-0.0005]	-0.0003 [-0.0026;0.0019]	0.0006* [0.005e-3;0.0012]
GDP Growth	0.0012 [-0.00002;0.0024]	0.0009 [-0.0004;0.0023]	0.0003 [-0.0007;0.0013]	0.0009 [-0.0013;0.0031]	0.0010* [0.0001;0.0020]	0.00002 [-0.0009;0.0009]	0.0007** [0.0002;0.0011]
**Model 3**							
Time trend (yearly)	0.0001 [-0.0013;0.0015]	0.0009 [-0.0013;0.0031]	0.0024*** [0.0017;0.0032]	0.0047* [0.0013;0.0080]	-0.0021*** [-0.0031;-0.0010]	-0.0002 [-0.0027;0.0023]	0.0007* [0.0001;0.0013]
Unemployment	0.0018 [-0.0015;0.0051]	-0.0010 [-0.0022;0.0002]	-0.0027** [-0.0047;-0.0008]	-0.0019 [-0.0051;0.0012]	-0.0015 [-0.0031;0.00004]	-0.0002 [-0.0019;0.0015]	-0.0008* [-0.0013;-0.0002]
**Model 4**							
Time trend (yearly)	0.0005 [-0.008;0.018]	0.00004 [-0.009;0.010]	0.0024*** [0.0015;0.0032]	0.0018 [-0.0010;0.0047]	-0.0019** [-0.0030;-0.0009]	0.0006 [-0.0028;0.0016]	0.0003 [-0.0003;0.0009]
Gini (income)	-0.0003 [-0.0028;0.0021]	-0.0065*** [-0.0089;-0.0041]	0.0005 [-0.0017;0.0027]	-0.0064 [-0.0141;0.0012]	0.0019 [-0.0003;0.0040]	-0.0027 [-0.0075;0.0021]	-0.0009 [-0.0022;0.0003]
**Model 5**							
Time trend (yearly)	0.0001 [-0.0013;0.0016]	0.0001 [-0.0017;0.0019]	0.0025*** [0.0017;0.0033]	0.0017 [-0.0020;0.0054]	-0.0014* [-0.0025;-0.0004]	-0.0005 [-0.0030;0.0021]	0.0008* [0.0001;0.0014]
GDP Growth	0.0016* [0.0003;0.0028]	0.0008 [-0.0002;0.0019]	0.0002 [-0.0007;0.0012]	0.0021 [-0.0004;0.0045]	0.0006 [-0.0003;0.0016]	0.0001 [-0.0011;0.0009]	0.0006* [0.0001;0.0010]
Unemployment	0.0034 [-0.0001;0.0068]	0.0003 [-0.0008;0.0014]	-0.0028* [-0.0049;-0.0007]	-0.0009 [-0.0040;0.0021]	-0.0020* [-0.0003;-0.0004]	-0.0004 [-0.0023;0.0016]	-0.0005 [-0.0011;-0.0001]
Gini (income)	-0.0006 [-0.0030;0.0019]	-0.0067*** [-0.0093;-0.0041]	-0.0002 [-0.0024;0.0020]	-0.0108* [-0.0190;-0.0025]	0.0024* [0.0002;0.0047]	-0.0028 [-0.0079;0.0022]	-0.0008 [-0.0020;0.0005]

Notes:

* Significant at p<0.05;

** significant at p<0.01;

*** significant at p<0.001.

CI Confidence interval

**Table 4:** Evolution of relative (RII) inequalities from 2005 to 2014, adjusting for GDP growth, unemployment, and Gini index.

	Scandinavian	Southern	Bismarckian	Anglo-Saxon	Post-communist	Former-USSR	Global
**RII** [95% CI]							
**Model 1**							
Time trend (yearly)	-0.0035 [-0.1054;0.1009]	-0.0199 [-0.0629;0.0230]	0.0344 [-0.0006;0.0694]	0.0614 [-0.0141;0.1369]	0.1112*** [0.0706;0.1519]	0.0150 [-0.0120;0.0420]	0.0313* [0.0063;0.0562]
**Model 2**							
Time trend (yearly)	0.0347 [-0.0604;0.1353]	-0.0266 [-0.0792;0.0259]	0.0385* [0.0027;0.0743]	0.0614 [-0.0168;0.1396]	0.1198*** [0.0747;0.1648]	0.0104 [-0.0169;0.0376]	0.0381** [0.0122;0.0641]
GDP Growth	0.1385** [0.0441;0.2330]	-0.0110 [-0.0595;0.0375]	0.0229 [-0.0197;0.0656]	-0.0019 [-0.0702;0.0663]	0.0168 [-0.0208;0.0543]	-0.0079 [-0.0188;0.0030]	0.0180 [-0.0019; 0.0380]
**Model 3**							
Time trend (yearly)	0.0477 [-0.0684;0.1631]	-0.0201 [-0.0568;0.0971]	0.0342* [0.0010;0.0674]	0.0626 [-0.0458;0.1711]	0.1107*** [0.0694;0.1521]	0.0117 [-0.0190;0.0424]	0.0466** [0.0190;0.0742]
Unemployment	-0.2400 [-0.5050;0.0251]	-0.0263 [-0.0684;0.0157]	-0.1178* [-0.2063;-0.0294]	-0.0016 [-0.1021;0.0988]	0.0077 [-0.0530;0.0684]	0.0051 [-0.0161;0.0263]	-0.0320* [-0.0580;-0.0061]
**Model 4**							
Time trend (yearly)	0.0076 [-0.0974;0.1125]	-0.0048 [-0.0455;0.0360]	0.0359 [-0.0008;0.0727]	0.0216 [-0.0659;0.1091]	0.1189** [0.0778; 0.1599]	0.0089 [-0.0176;0.0355]	0.0311* [0.0060;0.0561]
Gini (income)	0.1069 [-0.0936;0.3073]	-0.1473** [-0.2457-;-0.0489]	-0.0145 [-0.1119;0.0828]	-0.1884 [-0.4236;0.0469]	0.0667 [-0.0154;0.1488]	-0.0538 [-0.1109;0.0033]	-0.0072 [-0.0629;0.0484]
**Model 5**							
Time trend (yearly)	0.0963 [-0.0150;0.2076]	-0.0074 [-0.0835;0.0687]	0.0425* [0.0072;0.0777]	-0.0040 [-0.1376;0.1296]	0.124*** [0.0781; 0.169]	0.0058 [-0.0231;0.0347]	0.0493* [0.0211;0.0775]
GDP Growth	0.1302** [0.0338;0.2267]	-0.0157 [-0.0617;0.0304]	0.0182 [-0.0225;0.0589]	0.0395 [-0.0480;0.1271]	0.0089 [-0.0309;0.0487]	-0.0092 [-0.021;0.0023]	0.0123 [-0.0084; 0.0329]
Unemployment	-0.2048 [-0.4690;0.0593]	-0.0048 [-0.0495;0.0399]	-0.1283** [-0.2206;-0.0360]	0.0149 [-0.095;0.125]	-0.0063 [-0.0717;0.0591]	-0.0041 [-0.0259;0.0177]	-0.0278* [-0.0552;-0.0004]
Gini (income)	0.1945* [0.0074;0.3816]	-0.1441* [-0.2551;-0.0331]	-0.0479 [-0.1119;0.0475]	-0.263 [-0.557;0.0318]	0.064 [-0.0293;0.1584]	-0.0569 [-0.1139;4.62e-7]	0.0013 [-0.0545;0.0571]

Notes:

* Significant at p<0.05;

** significant at p<0.01;

*** significant at p<0.001.

CI Confidence interval

Adjusting for GDP or unemployment did not alter the significance of any of the time trend estimates for SII. The adjustment for Gini index and for all proxies taken together made the yearly trend non-significant in Anglo-Saxon countries. For RII, the adjustment for GDP growth, unemployment and all three proxies together brought statistical significance to the yearly trend in Bismarckian countries. The adjustment for covariates did not explain the time trend for post-communist countries.

GDP growth was positively associated with SII in Post-communist countries, and with RII in Scandinavian countries. Differently, unemployment was negatively associated with SII and RII in Bismarckian countries, and Gini index was negatively associated with SII and RII in Southern countries.

## Discussion

### Key findings

Our analysis shows that poor SRH decreased in Europe between 2005 and 2014, but its distribution remained concentrated among the least educated groups. Over the period, the highest RII values were mostly found in Post-Communist and Southern countries, surpassed in 2006, 2009, and 2012 by the Anglosaxon countries. As for SII, Post-Communist and Former-USSR countries showed the largest rate differences between least and most educated groups.

Over the 10-year period and for all countries taken together, absolute inequalities persisted and relative inequalities slightly widened. However, there were substantial differences across welfare regimes in the evolution of HI. Anglo-Saxon countries experienced the largest increase in absolute inequalities, followed by Bismarckian countries, while a statistically significant reduction was observed in Post-Communist countries. Post-Communist countries also experienced significant changes in relative inequalities, with a sharp widening trend across this period. In contrast, relative inequalities were stable in all remaining welfare regimes.

Finally, variations in GDP and unemployment did not explain any of the time trends in inequalities, and slightly obscured the rising trend in relative inequalities in Bismarckian countries. Variations on the Gini index turned the rising trend of absolute inequalities in Anglo-Saxon countries non-significant.

### Interpretation

The wide HI in this large population sample of 26 European countries is consistent with the evidence collected over time and locations.[3,4,8] The differences recorded across welfare regimes are partially in line with the results by Eikemo et al,[8] where Post-Communist, Former-USSR and Southern countries were among the welfare regimes with higher SII values, and Southern countries exhibited higher RII.

The apparent paradox of large absolute inequalities coupled with smaller relative inequalities in Post-communist and Former-USSR countries may be explained by the higher prevalence of poor SRH in these regions: the higher the prevalence of a certain condition, the higher the absolute differences between socioeconomic groups, and the lower the relative ones[3]. The different magnitudes of absolute inequalities between welfare regimes may be explained by their socioeconomic characteristics, as Former-USSR and Post-Communist countries have traditionally lower social protection expenditure as a percentage of GDP.[36,37]

The global trends of HI are in line with earlier studies by Kunst et al[4] and Hu et al,[3] confirming the persistence of HI over the last decades. However, the different patterns on the evolution across welfare regimes deserves further reflection. On the one hand, only Post-Communist countries presented a rise in relative inequalities, which confirms the trends previously described for Czech Republic, Poland, and Hungary.[3,15] On the other hand, the significant but small increases in absolute inequalities found in Bismarckian and Anglo-Saxon countries confirm some of the (non-significant) data from Hu et al.[3] Unexpectedly, Post-Communist countries showed a small narrowing trend of their absolute inequalities, which has not been previously noted in the literature. The remaining welfare regimes did not experience any significant change in the pattern of RII or SII, which goes in line with recent findings about the evolution of inequalities in SRH.[3,24]

The highest increase in absolute inequalities among Anglo-Saxon countries may be interpreted as the result of a more liberal welfare regime, and also of the response to the Great Recession. The substantial cuts to social protection, public health, and education services,[38,39] linked with the raise in income inequality may explain the rising absolute inequalities in Anglo-Saxon countries, during this period. In some Bismarckian countries, the recession followed by changes in labour market policies[24] and reduction of health budgets,[38,39] that may have led to the slight increase in absolute inequalities. These results seem to show that to prevent HI increasing during times of economic downturn, there is a minimum level to which the social safety net cannot go below.[13] Differently, in Post-Communist countries the prevalence of poor SRH decreased at a faster pace in the least educated group, leading to a reduction in absolute inequalities and an increase in relative ones. During this period, Post-Communist countries increased public spending on health, fostered government budget transfers to the health insurance scheme, and strengthened primary care and health promotion and prevention,[38,39] possibly with a higher effect on least educated groups. In comparison, Scandinavian and Former-USSR countries maintained a stable public health budget, despite the recession,[39] leading to stable HI. The stable trend in Former-USSR may also be linked to the fact that in these countries the public social spending is historically low and did not expressively change in the last decade.[40]

Surprisingly, and contrasting with previous studies[41], Southern countries, highly affected by substantial plunges in GDP, rises in unemployment rates, and under pressure to implement austerity measures, including sweeping public sector budget cuts, experienced stable trends in HI. This may be explained, firstly, by the characteristics of this welfare regime, where social support strongly relies on the family and charitable sector.[9] Secondly, budget cuts in healthcare mostly focused on renegotiations of drug expenditure[18] and corrections of longstanding sources of inequity in financing,[38] which may have actually contributed to reducing HI, instead of increasing them. Thirdly, in these countries the budget crisis was so extensive that it may have affected the population as a whole, and not only the least educated. Fourthly, unemployment particularly affected the youngest age groups, who are also the most resilient in terms of experiencing short term ill-health effects. In the same line, Spanish data has shown that good SRH has been improving at a faster pace among unemployed groups, and that unemployment during the economic crisis did not worsen SRH more than before it. [41,42]

Finally, we originally posited that trends in HI could be explained by economic cycles, proxied by variations in GDP, unemployment, and income inequality. However, our findings only confirmed this hypothesis amongst Anglo-Saxon countries, where rising absolute inequalities were explained by the growth in income inequality. Long-lasting and recently implemented counter-cyclical policies in some countries may have attenuated the effect of recession on the most vulnerable people.[18,38,39] It may well be also that GDP and unemployment rates, being only available at the national level, may have failed to capture geographical variations within countries, which may be more relevant to understand how people are affected by economic cycles.[43]

### Strengths and limitations

The analysis of the EU-SILC database allowed a comprehensive and recent overview of the evolution of HI. We studied the trends of HI among 26 European countries, analysing more than 3 million cross-sectional observations collected from 2005 to 2014, using socio-political and economic lenses. Also, this is one of the few studies that used comparable international data, collected using comparable methodology in all countries, over a 10-year period, and additionally adjusted using weighting methods to accurately reflect the population of each country.

Yet, some limitations must be considered. First, only countries with data from 2005 to 2014 were included, leaving Bulgaria, Croatia, Macedonia, Malta, Romania, Switzerland, Serbia and Turkey out of the analysis. Such limitation does not seem to have constrained the applicability of the results of this study, considering the size and variety of our sample. Second, we used as outcome a subjective measure of health. However, as referred before, SRH reflects both mental and physical health,[44] providing a more comprehensive and immediate perspective of population health than diagnosed conditions or mortality, and is widely considered a good predictor of health status and mortality.[22,44] In addition, this study focused on the time trend analysis of HI in SRH, not just on a one-off observation, which decreases the risk of bias due to cultural differences in health perception. Finally, in order to get a broad perspective on the evolution of HI in all these 26 European countries, we grouped them into six welfare regimes. Though the value of the ‘welfare state regimes approach’ is sometimes questioned[30,45], the typology grouping allows a theoretically-based analysis of the influence of socio-political and cultural characteristics of these countries on health outcomes, from the older theories of Esping-Andersen[46] and Ferrera,[27] to the more recent works of Bambra,[28] Aidukaite,[29] and Campos-Matos.[9] Moreover, welfare regimes have been considered important policy contexts in the aetiology of health inequalities[47] that should be taken into account in the study of the impact of debt crisis[5,11,48,49] and cannot be excluded from the explanation of the evolution of HI.[8,11,48,50]

## Conclusions

The study of the evolution of HI among 26 European countries revealed that, overall, HI persisted between 2005 and 2014, but this evolution varied across welfare regimes, possibly related to the varying social responses to the Great Recession and the underpinning levels of social protection offered. These results lend support to the hypothesis that consistent social investment and protection may help protect the population against the harmful effects of economic crises. Further studies are needed to create an evidence base for policies aimed at reducing HI in Europe.

## Supporting information

S1 Table Evolution of health inequalities between 2005 and 2014. Absolute (SII) and relative inequalities (RII) on poor SRH, globally, by social welfare regime and country (adjusted for sex and age).
(PDF)

S1 Fig Evolution of health inequalities between 2005 and 2014. Absolute (SII) and relative inequalities (RII) on poor SRH, globally and by social welfare regime (adjusted for sex and age).
(PDF)
