# Supplementary material for: Welfare states, the Great Recession and health: Trends in educational inequalities in self-reported health in 26 European countries
Source: PLoS One. 2018 Feb 23;13(2):e0193165. doi: 10.1371/journal.pone.0193165 (PMC5825059; doi:10.1371/journal.pone.0193165)
Supplement: S1 Table — Absolute (SII) and relative inequalities (RII) on poor SRH, globally, by social welfare regime and country (adjusted for sex and age). (PDF) [file pone.0193165.s001.pdf]

S1 Table: Evolution of health inequalities between 2005 and 2014. Absolute (SII) and relative inequalities (RII) on poor SRH, globally, by social welfare regime and country (adjusted for sex and age).

	2005	2006	2007	2008	2009	2010	2011	2012	2013	2014
**SII**										
**Scandinavian**	**0.058**	**0.062**	**0.058**	**0.053**	**0.053**	**0.054**	**0.061**	**0.053**	**0.057**	**0.064**
DK	0.032	0.034	0.033	0.046	0.032	0.054	0.061	0.044	0.044	0.047
FI	0.062	0.048	0.050	0.052	0.046	0.035	0.044	0.044	0.045	0.043
IS	0.051	0.054	0.072	0.039	0.039	0.060	0.084	0.061	0.084	0.081
NO	0.068	0.100	0.072	0.060	0.084	0.061	0.080	0.069	0.050	0.099
SE	0.062	0.067	0.056	0.050	0.056	0.063	0.054	0.034	0.061	0.055
**Southern**	**0.082**	**0.077**	**0.082**	**0.076**	**0.075**	**0.069**	**0.078**	**0.073**	**0.081**	**0.085**
CY	0.116	0.105	0.109	0.085	0.098	0.088	0.080	0.065	0.078	0.061
ES	0.086	0.075	0.077	0.056	0.057	0.057	0.049	0.053	0.054	0.065
IT	0.068	0.068	0.075	0.076	0.070	0.054	0.081	0.069	0.082	0.082
PT	0.118	0.115	0.120	0.150	0.146	0.146	0.147	0.136	0.141	0.151
**Bismarckian**	**0.073**	**0.074**	**0.073**	**0.078**	**0.083**	**0.082**	**0.088**	**0.083**	**0.082**	**0.094**
AT	0.082	0.071	0.072	0.086	0.085	0.089	0.09	0.092	0.084	0.105
BE	0.073	0.081	0.075	0.073	0.09	0.104	0.098	0.087	0.088	0.114
DE	0.067	0.084	0.070	0.093	0.10	0.098	0.116	0.096	0.104	0.119
FR	0.090	0.080	0.079	0.080	0.084	0.072	0.082	0.081	0.072	0.083
LU	0.087	0.082	0.088	0.096	0.087	0.088	0.100	0.097	0.122	0.108
NL	0.030	0.034	0.035	0.039	0.033	0.037	0.043	0.042	0.037	0.044
**Anglo-Saxon**	**0.059**	**0.062**	**0.052**	**0.046**	**0.059**	**0.053**	**0.063**	**0.101**	**0.086**	**0.075**
IE	0.030	0.037	0.024	0.024	0.036	0.022	0.031	0.036	0.039	0.040
UK	0.078	0.078	0.070	0.058	0.075	0.075	0.082	0.135	0.120	0.100
**Post-communist**	**0.176**	**0.170**	**0.174**	**0.168**	**0.163**	**0.158**	**0.161**	**0.160**	**0.159**	**0.165**
CZ	0.161	0.159	0.168	0.155	0.160	0.159	0.164	0.174	0.145	0.159
HU	0.218	0.210	0.221	0.200	0.181	0.174	0.181	0.184	0.190	0.205
PL	0.164	0.151	0.151	0.153	0.159	0.147	0.134	0.133	0.135	0.138
SI	0.200	0.183	0.192	0.196	0.175	0.162	0.171	0.161	0.163	0.166
SK	0.149	0.160	0.170	0.148	0.133	0.150	0.166	0.151	0.147	0.159
**Former-USSR**	**0.151**	**0.153**	**0.154**	**0.164**	**0.156**	**0.150**	**0.167**	**0.141**	**0.158**	**0.146**
EE	0.123	0.142	0.143	0.131	0.139	0.134	0.147	0.121	0.145	0.120
LT	0.151	0.158	0.154	0.178	0.173	0.153	0.203	0.186	0.191	0.184
LV	0.182	0.163	0.171	0.180	0.151	0.154	0.148	0.121	0.144	0.142

S1 Table: (continuation)

	2005	2006	2007	2008	2009	2010	2011	2012	2013	2014
**RII**										
**Scandinavian**	**2.320**	**2.525**	**2.762**	**2.540**	**2.611**	**2.634**	**2.706**	**2.614**	**2.628**	**2.832**
DK	1.770	1.738	1.692	2.100	1.753	2.475	2.343	1.877	1.929	1.957
FI	2.179	1.873	2.445	2.567	2.372	1.885	2.119	2.196	2.436	2.493
IS	2.536	4.796	9.909	3.278	2.353	3.932	3.544	3.980	4.456	3.613
NO	2.402	3.591	3.000	2.382	3.548	2.498	3.012	3.376	2.005	3.437
SE	2.658	3.222	3.306	2.595	2.942	4.147	3.419	2.537	3.705	3.035
**Southern**	**2.900**	**2.700**	**2.851**	**3.174**	**2.982**	**2.928**	**3.016**	**2.857**	**3.020**	**2.946**
CY	4.917	4.056	3.842	3.752	3.741	4.038	3.465	3.387	3.702	3.377
ES	2.648	2.416	2.573	2.919	2.777	2.899	2.723	2.753	2.570	2.889
IT	2.830	2.789	2.956	3.084	2.821	2.421	2.799	2.536	3.043	2.976
PT	2.782	2.729	2.948	4.633	4.055	3.664	4.577	3.756	3.885	3.097
**Bismarckian**	**2.498**	**2.659**	**2.643**	**2.957**	**3.097**	**2.969**	**3.064**	**3.022**	**2.911**	**3.357**
AT	3.473	3.334	2.847	3.035	2.925	3.298	2.950	3.603	2.856	3.760
BE	2.821	3.288	3.111	3.007	3.194	3.684	3.261	2.987	3.196	3.801
DE	1.931	2.267	2.035	2.809	3.156	2.905	3.154	2.779	3.024	3.484
FR	3.318	3.034	3.323	3.401	3.188	2.790	3.163	3.029	2.783	3.229
LU	4.523	5.566	5.619	5.573	3.899	4.251	5.086	5.272	5.368	4.422
NL	2.054	2.381	2.214	2.412	2.425	2.367	2.590	2.620	2.306	2.793
**Anglo-Saxon**	**2.882**	**3.565**	**2.917**	**2.680**	**3.503**	**2.925**	**3.438**	**4.205**	**3.707**	**3.141**
IE	2.264	3.143	2.363	2.266	3.307	2.069	2.535	3.043	2.694	2.997
UK	3.101	3.727	3.072	2.795	3.561	3.315	3.817	4.486	4.169	3.197
**Post-communist**	**2.824**	**2.846**	**3.028**	**3.090**	**3.147**	**3.132**	**3.341**	**3.450**	**3.443**	**3.840**
CZ	3.353	3.762	4.847	3.928	4.404	4.176	4.418	5.743	4.051	4.888
HU	3.179	2.941	2.908	3.053	2.972	2.992	3.296	3.288	3.510	4.190
PL	2.569	2.579	2.721	2.667	2.861	2.789	2.751	2.698	2.659	2.951
SI	4.258	3.977	4.167	4.623	4.028	3.581	3.752	4.485	5.262	5.403
SK	2.382	2.298	2.508	2.575	2.579	3.111	3.664	3.784	3.716	3.769
**Former-USSR**	**2.296**	**2.340**	**2.399**	**2.572**	**2.556**	**2.417**	**2.562**	**2.266**	**2.618**	**2.400**
EE	2.083	2.551	2.505	2.424	2.719	2.417	2.309	1.969	2.541	2.169
LT	2.494	2.375	2.446	2.655	2.604	2.434	3.135	2.687	2.801	2.841
LV	2.298	2.121	2.320	2.606	2.386	2.366	2.330	2.185	2.537	2.307
