# Supplementary material for: Welfare states, the Great Recession and health: Trends in educational inequalities in self-reported health in 26 European countries
Source: PLoS One. 2018 Feb 23;13(2):e0193165. doi: 10.1371/journal.pone.0193165 (PMC5825059; doi:10.1371/journal.pone.0193165)
Supplement: S1 Fig — Absolute (SII) and relative inequalities (RII) on poor SRH, globally and by social welfare regime (adjusted for sex and age). (PDF) [file pone.0193165.s002.pdf]

**SII**											
	2005	2006	2007	2008	2009	2010	2011	2012	2013	2014	2005-2014
Scandinavian	0,058	0,062	0,058	0,053	0,053	0,054	0,061	0,053	0,057	0,064	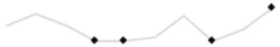
Southern	0,081	0,076	0,081	0,076	0,076	0,07	0,078	0,073	0,079	0,084	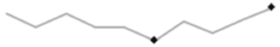
Bismarckian	0,073	0,074	0,073	0,078	0,083	0,082	0,088	0,083	0,082	0,094	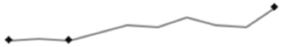
Anglo-Saxon	0,059	0,062	0,052	0,046	0,059	0,053	0,063	0,101	0,086	0,075	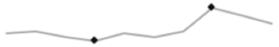
Post-Communist	0,176	0,17	0,174	0,168	0,163	0,158	0,161	0,16	0,159	0,165	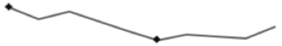
Former-USSR	0,151	0,153	0,154	0,164	0,156	0,15	0,167	0,141	0,158	0,146	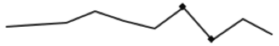
Global	0,0996	0,0997	0,102	0,0997	0,1011	0,097	0,1049	0,101	0,1024	0,1058	

**RII**											
	2005	2006	2007	2008	2009	2010	2011	2012	2013	2014	2005-2014
Scandinavian	2,32	2,525	2,762	2,54	2,611	2,634	2,706	2,614	2,628	2,832	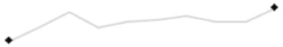
Southern	2,946	2,719	2,92	3,189	3,057	2,983	3,004	2,879	2,919	2,888	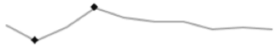
Bismarckian	2,498	2,659	2,643	2,957	3,097	2,969	3,064	3,022	2,911	3,357	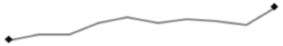
Anglo-Saxon	2,882	3,565	2,917	2,68	3,503	2,925	3,438	4,205	3,707	3,141	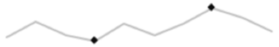
Post-Communist	2,824	2,846	3,028	3,09	3,147	3,132	3,341	3,45	3,443	3,84	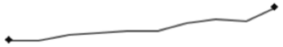
Former-USSR	2,296	2,34	2,399	2,572	2,556	2,417	2,562	2,266	2,618	2,4	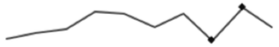
Global	2,556	2,607	2,709	2,826	2,922	2,8414	2,963	2,9366	2,965	3,0715	

S1 Figure: Evolution of health inequalities between 2005 and 2014. Absolute (SII) and relative inequalities (RII) on poor SRH, globally and by social welfare regime (adjusted for sex and age).
